# Microporous Zr-metal-organic frameworks based-nanocomposites for thermoelectric applications

**DOI:** 10.1038/s41598-024-62317-3

**Published:** 2024-06-06

**Authors:** Asmaa Ebrahim, Mohsen Ghali, A. A. El-Moneim

**Affiliations:** 1 School of Basic and Applied Science, Egypt-Japan University of Science and Technology, New Borg El Arab City, Alexandria, 21934 Egypt; 2 Graphene Center of Excellence for Energy and Electronic Applications, Egypt-Japan University of Science and Technology, New Borg El Arab City, Alexandria, 21934 Egypt; 3 Physics Department, Faculty of Science, Kafrelshiekh University, Kafr el-Sheikh, Egypt

**Keywords:** Thermoelectric, Seebeck coefficient, Hall coefficient, In-situ polymerization, Conductive MOFs, Conductive polymer, Chemistry, Materials chemistry, Polymer chemistry, Materials science

## Abstract

In the area of energy storage and conversion, Metal-Organic Frameworks (MOFs) are receiving more and more attention. They combine organic nature with long-range order and low thermal conductivity, giving them qualities to be potentially attractive for thermoelectric applications. To make the framework electrically conductive so far, thermoelectricity in this class of materials requires infiltration by outside conductive guest molecules. In this study, an in-situ polymerization of conductive polyaniline inside the porous structure of MOF-801 was conducted to synthesize PANi@MOF-801 nanocomposites for thermoelectrical applications. The growth of polyaniline chains of different loadings inside the host MOF matrix generally enhanced bulk electrical conductivity by about 6 orders of magnitude, leading to Seebeck coefficient value of -141 µVK^−1^ and improved thermal stability. The unusual increase in electrical conductivity was attributed to the formation of highly oriented conductive PANi chains inside the MOF pores, besides host–guest physical interaction, while the Seebeck coefficient enhancement was because of the energy filtering effect of the developed structure. Modulating the composition of PANi@MOF-801 composites by varying the aniline: MOF-801 ratio in the synthesis bath from 2:1 and 1:1 to 1:2 leads to a change in the semiconductor properties from p-type semiconductor to n-type. Among the examined composites with n-type semiconducting properties exhibited the highest ZT value, 0.015, and lowest thermal conductivity, 0.24 Wm^−1^ K^−1^. The synthesized composites have better performance than those recently reported for a similar category of thermoelectric materials related to MOF-based composites.

## Introduction

Thermoelectric (TE) devices turn heat into electricity or vice versa without mechanical parts, making them ideal for harvesting energy and cooling systems^1–3^. The efficiency of a TE device is determined by the material figure of merit, ZT = S^2^σT/(k_e_ + k_L_), where T is the absolute temperature, S is the Seebeck coefficient, σ is the electrical conductivity and k_e_ and k_L_ are the electronic and lattice thermal conductivities, respectively. The ZT of commercial TE materials (such as Bi_2_Te_3_) is close to one, and there is no theoretical upper limit to ZT^4–6^. Accordingly, discovering low-cost, high-performance, and environmentally friendly TE materials with high zT to accelerate the commercialization of TE devices represents a global demand^7–9^. Owing to their advantageous functional properties and topologically diverse architectures, porous metal–organic frameworks, or MOFs, have recently become an important class of materials^3^. An optimal platform for a multitude of potential applications is provided by their rich coordination chemistry, high porosity, and ease of surface tunability^3^. Intrinsically occurring voids and pores in the structure MOFs act as barriers for phonon transport, reducing lattice thermal conductivity k_L_ and electron tunneling. Besides, the electrical properties of MOFs are halted due to the high energy barrier for electron transfer caused by the minimal overlap between the ligands’ π-orbitals and the metal ions' d-orbitals in porous MOFs. In theory, MOFs with exceptionally high electrical and low thermal conductivity, hence high thermoelectric properties, can be created by proposing a new structural design that maintains the advantage of diversified composition and high porosity^10,11^. Despite several trials conducted recently to enhance the electrical conductivity of MOFs^12,13^ through utilizing metal nodes with high-energy electrons^14,15^ or redox-active linkers^16^ or organic^18,19^ or inorganic doping^17^ species. Nonetheless, the reported research fulfilled the requisites for practical applications for this category of materials. As a result, it is vital to investigate multiple synthetic routes to maximize electrical conductivity and increase the family of conductive 3D MOFs^20^. In this regard, Allendorf and coworkers increased the electrical conductivity of Cu3(BTC)2 MOF by six orders of magnitude by infiltrating its nanopores with conductive 7,7,8,8- tetracyanoquinododimethane^21^. Meanwhile, Zue et al., incorporated a tetrathiafulvalenium (TTF^.+^) and N,N′-dimethyl-4,4′-bipyridinium (MV^2+^) radicals into (Me2NH2)[In^III^(TTFTB)].0.7C2H5OH.DMF MOF via cation exchange resulted in the formation of (TTF^.+^) [In(TTFTB)].5DMF (MV^2+^)0.5[In(TTFTB)].2DMF structure with improved electrical and photoelectron conductivity^22^. Kitagawa and co-workers polymerized 3,4-ethylenedioxythiophene in MIL-101(Cr) pores to obtain a conductive composite with an electrical conductivity of 1.1*10^–3^ Scm^−1^^23^. Li et al., reported the preparation of composite film from PEDOT: PSS@HKUST-1 with electrical conductivity 13 Scm^−1^, which is 9 orders of magnitude enhanced electrical conductivity than pristine HKUST-1 (Cu3(BTC)2)^24^. Such activities opened the routes for further developments of conductive MOFs and their potential application in thermoelectric studies using conductive polymers. The conductive polymer polyaniline (PANi) is widely used in flexible energy harvesting systems because of its low toxicity, environmental stability, low production cost, and lightweight design. However, its inherited low thermal conductivity, Seebeck coefficient, along with low electrical conductivity in the range of 10^−7^–3 × 10^2^ Scm^−1^ has resulted in low PF 10^−6^–10^−10^ Wm^−1^ K^−2^ hindering the extensive application of PANi in TEG^7,8,12,24^. Zr-MOFs comprise a unique class of structurally diverse MOFs with excellent water stability^25^. Among them, MOF-801 has a formula of Zr_6_O_4_(OH)_4_(fumarate)_6_ and Face centered cubic (fcu) topology with plenty of hydroxyl groups involved in the nodes of Zr(IV) that facilitate the acceptance of guest molecules in its pores for different applications. Also, the different sizes of pore diameters in MOF-801 (0.48, 0.56, 0.74 nm) can effectively scatter phonons that reduce the lattice’s thermal conductivity^26^. Combining PANi and MOF-801 with an efficient synthesis methodology without changing the MOF primary structure can be presented as a new generation of TE materials worthy of exploration. For instance, UiO-66/PANI/PSS composite film prepared from polyaniline thread-entangled UiO-66 composite blended with polystyrene sulfonate as a dopant achieved an extremely large negative Seebeck coefficient of −17,780 µVK^−1^ with a high power factor of 664 μW m^−1^ K^−2^^24^. It is believed that the phase junction due to structurally stable porous frameworks derived from MOFs and the homogeneously immobilized conducting PANi would allow phonon scattering, producing hybrid systems with low thermal conductivity and enhanced electrical conductivity. Till now, very few reported studies offer full thermoelectric characterizations for the produced polymer @ MOFs composites, such as hall coefficient, carrier concentration and mobility, electrical and thermal conductivities, and Seebeck coefficient related to the loaded polymer percentages and their subsequent energy-filtering effect. As a result, detailed investigations that consider all thermoelectric parameters still need to be illustrated to better understand the thermoelectric properties of guest @ MOFs composites.

We are aware that there are no publications on synthesizing PANi-based MOF (MOF-801) composite materials for thermoelectric applications. The hydrothermal method was used to create the MOF-801 because it offers precise control over size, shape distribution, well-crystallized nanoscale solids, effective synthetic conditions, and the ability to regulate the polymer-to-MOF ratio. The final PANi@MOF-801 composites demonstrated promising thermoelectric material behavior. This work focuses on key strategies in developing high-performance PANi @ MOFs composites for room temperature thermoelectric application. The energy-filtering effect, and interface engineering add interfacial interactions to TE composites. The overall performance of the customized TE composites has been investigated for sustainable energy utilization with appropriately optimized components, microstructure, and fabrication processing, which help in the carrier transport properties.

## Experimental

### Materials

Zirconium oxychloride octahydrate (ZrOCl_2_·8H_2_O, purity ≥ 99.8%), *N*, *N*- dimethylformamide (DMF) (HPLC grade), fumaric acid (purity ≥ 99%), formic acid, methanol, ammonium persulphate (APS,99%), acetone, Aniline, HCl. All chemicals are used as received without any treatment or further purification.

### Synthesis of MOF-801, Zr_6_O_4_(OH)_4_(fumarate)_6_

**Figure 1:**
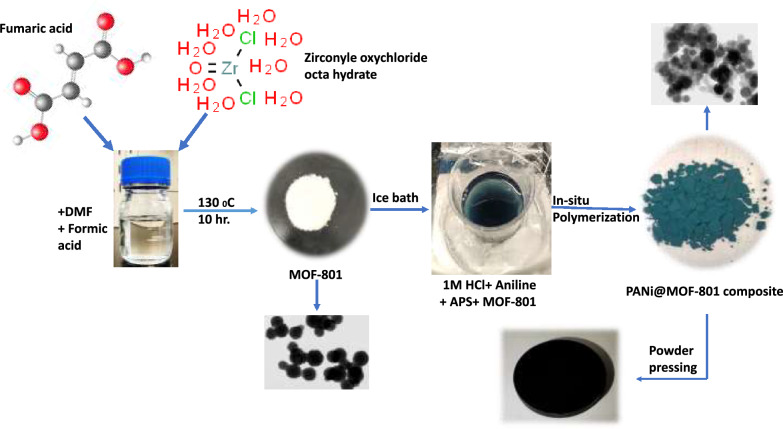
Schematic illustration of the preparation of MOF-801 and Polyaniline nanocomposites.

MOF-801 was synthesized by the method reported in detail elsewhere^25^. In a mixture of DMF (40 ml) and formic acid (14 ml), 50 mmol of fumaric acid, and 50 mmol of ZrOCl_2_·8H_2_O were dissolved. The mixture was placed in a 100 ml screw-capped hydrothermal jar that was then heated in an oven at 130 °C for 10 h to produce a white precipitate. The collected precipitate was centrifuged and washed with DMF and methanol. Finally, the collected powder is dried in a vacuum oven at 100 °C overnight, Fig. 1.

### Synthesis of PANi@MOF-801

A certain amount of MOF-801 was ultrasonically dispersed in a solution of 4 mmol ammonium persulphate (APS) in 20 ml of 1M HCl for 30 min. Another solution of 4 mmol of the monomer aniline was dissolved in 20 ml 1M HCl with stirring until a yellowish-clear solution was obtained. In an ice bath (< 10 °C), the aniline solution was added drop by drop to the MOF solution, allowing the in-situ polymerization of polyaniline for 4 h. The prepared powder with a dark green colour (emeraldine salt) was collected after centrifugation, washed with distilled water/ethanol, and dried in a vacuum oven at 50 °C overnight. For the preparation of pristine polyaniline (PANi), the same procedure was followed in the absence of MOF addition. Three different PANi@MOF-801 composites were prepared based on aniline: MOF-801 mixing ratios of 2:1, 1:1, and 1:2 (0.4:0.2, 0.4:0.4, and 0.4:0.8 wt/wt, respectively) to understand the role of the individual composite components on the recorded thermoelectric properties. The samples were then cold pressed into pellets with a diameter (13 mm) and thickness (2 mm) using a vacuum hydraulic presser under pressure (100 kPa) for 5 min at room temperature for thermoelectric characterization, Fig. 1.

### Physicochemical characterizations

X-ray powder diffraction (PXRD) data were collected on an X-ray 7000 Shimadzu-Japan using Cu Kα radiation (λ = 1.5406 Å) generated at 30 kV and 30 mA at varying 2θ from 3° to 70° with a step width of 5 min^−1^. Fourier-transform infrared spectra (FTIR) were collected from FTIR; Bruker, Vertex 70 spectrophotometer with KBr pellets at a wavelength ranging from 400 to 4000 cm^−1^ with a resolution of 4 cm^−1^. Morphological features and compositional analysis were performed by using a scanning electron microscope SEM complemented with energy-dispersive spectroscopy (EDX) (SEM Quanta FEG 250) and transmission electron microscopy (TEM; JEOL JEM-2100F). The thermal stability of MOF-801 and PANi@MOF-801 composite was featured via thermogravimetric analysis (TGA) using TG-DSC Labsys 1600C Setaram, where the weight loss was measured at a temperature range of 25–700 °C under N_2_. The specific surface area (SSA) and porosity were measured by Brunauer–Emmett–Teller (BET) with a Microtrac MRB instrument, using N_2_ as the adsorbate at − 19 °C. Zeta potential measurements of surface charge were made on a Malvern Zetasizer Nano-ZS (Malvern Instruments)^7^.

### Thermoelectric measurements

The thermal conductivity was measured using thermal analyzer (Hot Disk TPS 2500s), where a 7577 Kapton-coated sensor that serves as both a heat source and a thermometer was positioned between two identical samples^7^. The input power and time were adjusted accordingly to stop heat from spreading outside the sample. The electronic properties of the tailored composites, including carrier mobility and concentration, and electrical conductivity were measured at room temperature by an Ecopia 7000 Hall effect measurement system with a magnetic field of 0.51 T and an electric current of 1 mA, at room temperature, where the contact for electrical connections was established using Ag-paste. The Seebeck coefficient of the pellets was measured using a home-built steady-state measurement system^7^. In this measurement the sample (pellet with diameter of 12 mm and thickness of 2 mm was sandwiched between the copper heater and a Teflon block on the cold side. To reduce the electrical and thermal contact resistances, the sample was mounted on the graphite layer. The heater assembly is comprised of a small copper block with a cross-sectional shape and size similar to the sample, and an electrical heater brazed into it. The wire leads for the current and voltage as well as a K-type thermocouple are also integrated into the hot junction assembly. To reduce radiative heat loss, a copper radiation shield encased the entire assembly. The Seebeck coefficient (S) was computed as S = ΔV/ΔT after heating the copper block and recording the thermovoltage change (ΔV) as a function of the temperature difference (ΔT) between the contacts^7^. Diffuse reflectance spectroscopy (DRS) was utilized to determine the band gap of the prepared composites (V-770-JASCO NIR Spectrophotometer).

## Results and discussion

### Morphological and structural characterization of MOF-801 and PANi@MOF-801

**Figure 2:**
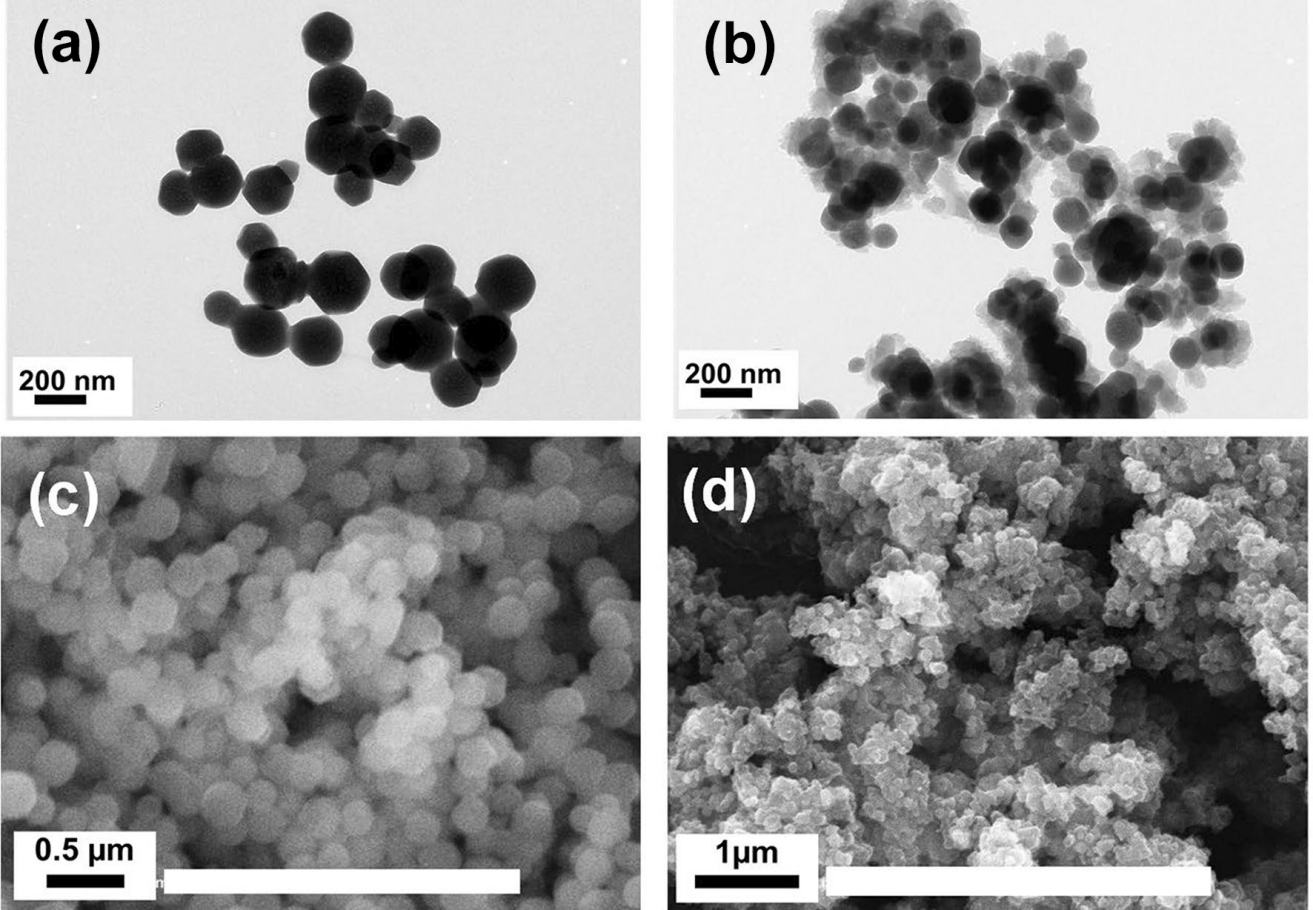
TEM images for MOF-801 (**a**) and PANi@MOF-801 (2:1) composite (**b**). SEM images for MOF-801 (**c**) and PANi@MOF-801 (2:1) composite (**d**).

Figure 2a–d elucidates SEM and TEM images for MOF-801 and PANi@MOF-801 (2:1) composite. The images for pristine MOF-801 reveal the formation of hexagonal crystals with an average particle size of 160 nm, as published before in other work^26^. The images of PANi@MOF-801(2:1) composite, reveal a wrapping (interweaving) of the MOF-801 crystals surface by in-situ polymerized PANi platelets. Moreover, the TEM image in Fig. 2b suggests that the in-situ polymerization of PANi happened on the surface and inside the MOF pores, which caused the stretching of the polymer chains with changes in the morphology and the degree of crystallinity of the polymerized PANi and consequently, enhanced electrical properties.

**Figure 3:**
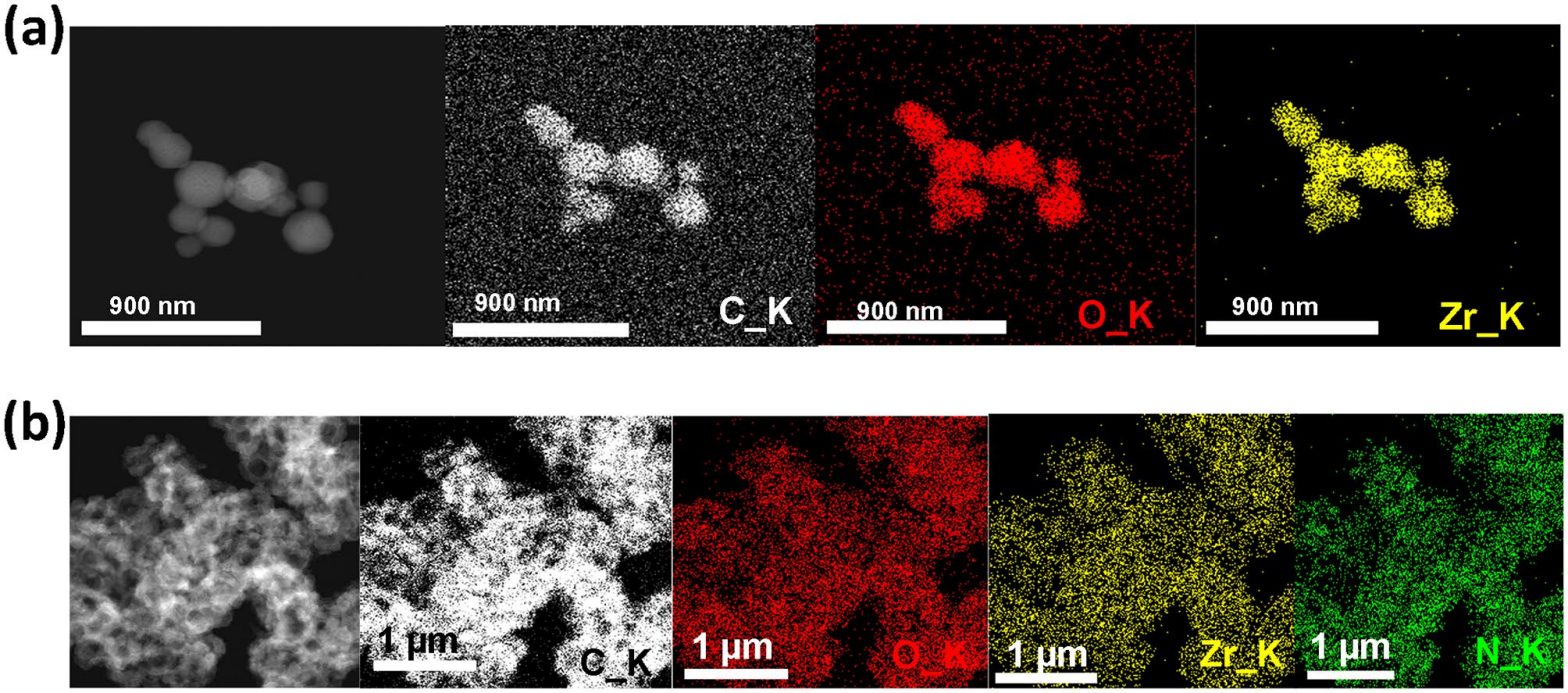
EDXS mapping for; (**a**) MOF-801 and (**b**) PANi@MOF-801.

Energy dispersive X-ray spectroscopy (EDXS) analysis shows the existence of stoichiometric distribution of C, O, N (N originates from PANi) and Zr elements through the surface and voids of PANi@MOF-801 sample, Fig. 3.

**Figure 4:**
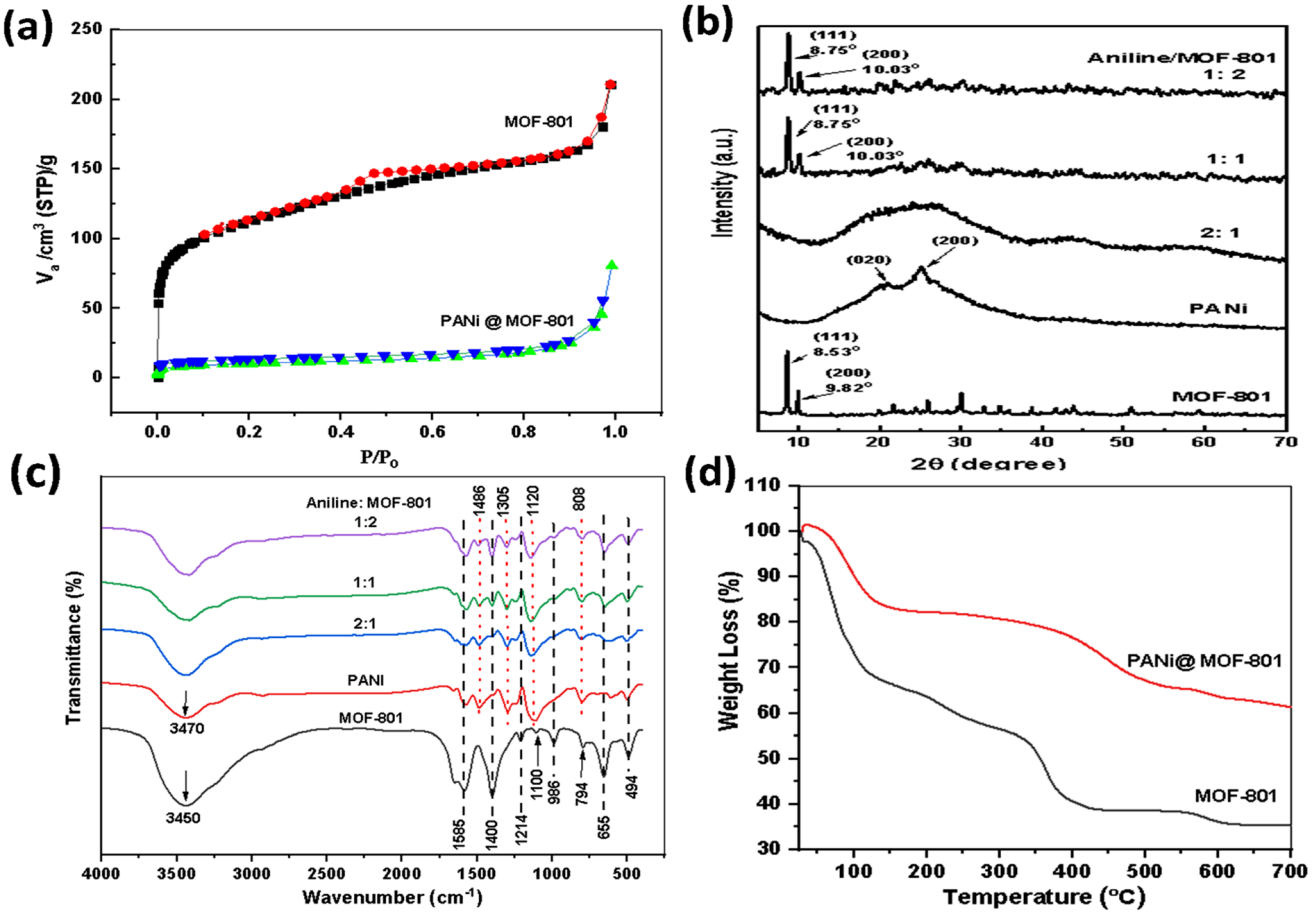
(**a**) BET isotherm, (**b**) PXRD, (**c**) FT-IR, (**d**) TGA of; (MOF-801, PANi, and the composites of PANi @ MOF-801 composites.

The surface area and porosity of desolvated MOF-801 and PANi@MOF-801(2:1) samples were characterized by BET analysis at 77 K by using N_2_ as probe gas, Fig. 4a. The N_2_ adsorption–desorption isotherms of both samples confirm the microporous structure of the investigated samples. The calculated BET-specific surface area, total pore diameter and volume of MOF-801 are 550 m^2 ^g^−1^,11.3 Å and 0.245 cm^3^ g^−1^, which is consistent with other groups working with the same MOF (750 m^2^ g^−1^ found by Mohammed and coworkers^27^;680 m^2 ^g^−1^ reported by Serre et al.^28^), while those for PANi@MOF-801 (2:1) are found 36.4 m^2 ^g^−1^, 8.5 Å and 0.121 cm^3^ g^−1^, respectively. Such reduction of surface area, pores diameter, and volume can be ascribed to the partial blockage of MOF pores by PANi. Also, the isotherms of MOF-801 and PANi@MOF-801 present a striking gas uptake between the relative pressures of 0.9 and 1.0. Such uptakes indicate that filling the gaps between MOF nanocrystals by PANi is minimal, and a highly oriented growth of PANi chains inside these micropores is understandable from the fact that the dimension of aniline monomers of the same order of the pore volume of MOF-801.

To check the influence of PANi inclusion on the crystallinity of PANi@MOF-801 composites, we have performed PXRD analysis on PANi and MOF-801, and PANi@MOF-801 composites with different aniline: MOF-801 ratios, Fig. 4b. The diffraction pattern for MOF-801 agrees with both the pattern simulated from single-crystal X-ray data and the data from the literature, where it shows peaks located at 2θ = 8.65, 10.01, 13.9, 19.9, and 21.7° indexed to (111), (200), (222), (420), and (440) plans. The cubic space group fcu topology may be used to index these patterns with an octahedral cavity and two tetrahedral cavities^25^. The calculated average crystallite size of 27 nm and degree of crystallinity of 70% confirming the genuine structure of the synthesized MOF-801. The pattern of neat PANi exhibited a major diffuse amorphous peak at 2θ between 11° and 37° with two minor crystalline peaks emerging at 2θ of 20.86° and 25.08° indexed to (020) and (200) planes, respectively^25^. These minor peaks are due to quinonoid and benzenoid groups in the PANi backbone with a crystallinity of 48%, indicating the amorphous nature of the polymer^29^. The diffraction pattern of PANi@MOF-801(2:1) shows only a diffuse peak typical of the amorphous structure. The amorphous nature of PANi overcomes the crystallinity of the MOF, and the whole PXRD pattern has a wide broad diffraction peak between 2θ = 12.7°–38° that is close to that of pristine PANi broad peak but with a slight shift may be due to the presence of MOF-801. This finding revealed that PANi’s amorphous structure was preserved during the composite’s formation with MOF. The same pattern was noticed in the PANi/Cu-trimesic (HKUST-1) composite for hydrogen production applications. The main diffraction peaks of the MOF disappeared in the composite XRD pattern^30^. By modifying the aniline/MOF ratio in the synthesis bath from 2:1 to 1:1 and 1:2, the recorded XRD patterns in PANi@MOF-801 composites well-reserved the MOF-801 structure and the amorphous part, characteristic of PANi, was not present in the PANi@MOFs patterns. A blue shift modulation in the 2θ values of MOF major peaks and the appearance of two new minor crystalline peaks at 2θ of 20.86 and 25.08° are noted, which got slightly sharper with increasing MOF content. The appearance of some new peaks and peak modulation could originate from the growth of repeating units of PANi inside the pores and channels of the MOF-801 framework. This indicates the interaction between the composite constituents and the crystallinity of PANi was enhanced when Aniline was in situ polymerized within the MOF-801 framework.

Fourier transform infrared spectroscopy was also conducted on PANi, MOF-801, and PANi@MOF-801 composites to verify that PANi was successfully merged with the MOF-801, Fig. 4c. The FT-IR spectrum of MOF-801 has characteristic bands at 494, 1100, 1214, 1400, 1585, 3450, 986, 655, and 794 cm^−1^. These bands belong to the Zr–O stretching of the central Zr-cluster, the –C=O–OH and –C–O from the functional group of the fumaric acid linker, and the asymmetric and symmetric vibrations of the –COOH group, O–H stretching vibration of carboxylate, CH_3_ skeletal vibrations, Zr_6_(OH)_4_O_4_ vibrations, and C=C–H out-of-plane bending, respectively^25–27^. The characteristic PANi absorption bands occur at 3470, 1576.6, 1486, 1305.7, 1120, 3462.1, and 808.3 cm^−1^, which are due to the N–H asymmetric stretching, Quinoid, Benzenoid rings, C–N stretching, C–H in-plane bending vibrations, N–H stretching vibrations, and C–H out-of-plane bending vibrations, respectively^28,29^. The in-situ polymerization of PANi in MOF-801 pores led to the appearance of two bands, typical characteristics of PANi at 1120 and 1486 cm^−1^, in the spectrum of MOF-801. The intensity of these bands is reduced, and their position slightly shifted to a higher frequency as the content of aniline compared to MOF-801 in the synthesis bath is decreased, confirming a possible π–π/N–H–π interaction/or charge transfer interactions between the host backbone and guest moieties. Also, the disappearance of other peaks from pure PANi and MOF-801 in the composites’ FT-IR spectra is due to the in-situ polymerization and the combination effect of both materials. The thermal stability of MOF-801 and PANI@MOF-801(2:1) samples was explored using TGA characterization technique, Fig. 4d. For pristine MOF-801, the TGA curve shows weight loss in three stages up to about 400 °C. The sample's initial weight loss of about 30% happened below 125 °C and is explained by the moisture being removed from it. The subsequent evaporation of solvent guest molecules between 125 and 350 °C resulted in an additional 25–30% weight loss. The sample began to collapse when the temperature was raised above 350 °C, and it broke down entirely at about 400 °C because of the carboxylate groups in the network breaking^27^. Incorporating PANi with MOF-801 enhances the MOF thermal stability at higher temperatures. It reduces the mass loss rate with increasing temperature, further confirming the effective bonding between the MOF and the polymer chains in PANi that gives higher thermal stability of the composites at elevated temperatures. Furthermore, the decomposition steps observed in the TGA curves in the case of the polymer @ MOF composites showed slower and less steep degradation steps compared to that of pristine MOF-801. This lower rate of weight loss at higher temperatures suggested a good combination and effective bonding between the MOF and the polymer chains.

**Figure 5:**
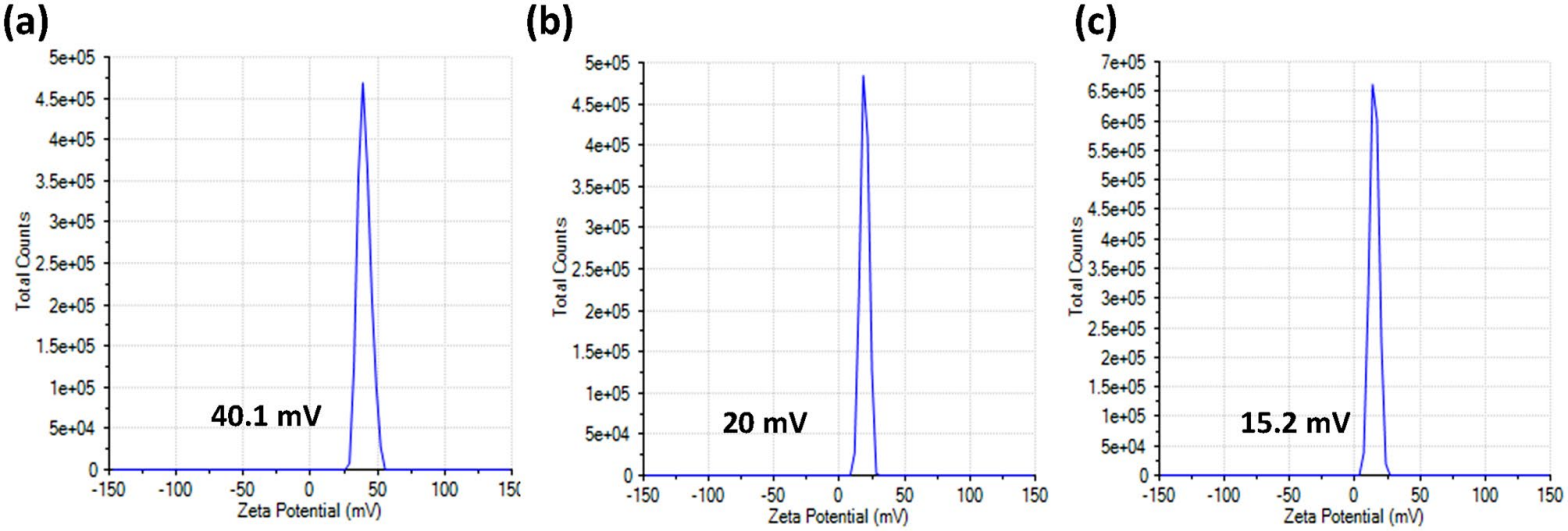
Zeta potential of; (**a**) MOF-801, (**b**) PANi, (**c**) PANi@MOF-801(1:2).

The surface charge of MOF-801, PANi, and PANi @MOF-801 (2:1) was measured in water (pH = 6–7) at room temperature, Fig. 5a–c, and show positive values of 40.1, 20 and 15.2 mV, respectively. Such high positive values for both MOF and PANi enable good dispersity and stability of the MOF and PANi in water. This would allow a good distribution of the polymer chains inside the framework and among particles of the MOF in the polymerization media. The positive surface charges of both MOF and PANI could be the result of the amorphous XRD pattern, Fig. 4b, with an Aniline/MOF ratio of 2:1 due to the electrostatic repulsion between the two surfaces which prevents the formation of the homogeneous composite.

This effect could be proved from TEM and SEM images of this composite Fig. 2b, d. The composite showed a reduced zeta potential value of 15.2 mV, allowing the aggregation of the particles as indicated by the TEM images, Fig. 2c.

In brief, the results support the conclusion that the in-situ polymerized PANi was successfully interpenetrated into the MOF-801 voids with chain-like crystalline structures. The PANi would also warp or interweave the MOF nanocrystals through electrostatic interaction, as evidenced by TEM images, PXRD, and FTIR analysis. Such change in structural topologies of MOF by in-situ polymerization of PANi would offer versatile regulation strategies for creating conductive pathways, pores^26^, and structural long-range crystalline order, which can be harnessed to promote charge transfer, phonons scattering, and energy filtering and thus be anticipated in pursuing higher ZT.

### Thermoelectric properties of PANI@MOF-801 nanocomposites

### Thermoelectric properties

The thermopower characteristics, electrical (σ)/thermal (κ) conductivity, and Seebeck coefficient (S) were investigated in terms of tuning the composition of the PANi@MOF-801 composites for the ideal thermoelectric performance. In principle, unlike conducting polymers, MOFs are poor electrical conductors for carriers. This is due to the presence of insulating organic linkers, which form the framework's backbone, and the lack of orbital overlap between the metal center's d orbitals and the s/p orbitals of the ligands utilized^31^. Efforts have been undertaken in recent years to effectively convert these insulating materials into semiconducting ones using various tactics such as the intrinsic and extrinsic approaches^11^. The latter situation can be accomplished by inserting conducting guest molecules, such as PANi, PPy, and PEDOT, into the MOF framework’s voids^23,24,29–32^.

#### Electrical transport properties

**Figure 6:**
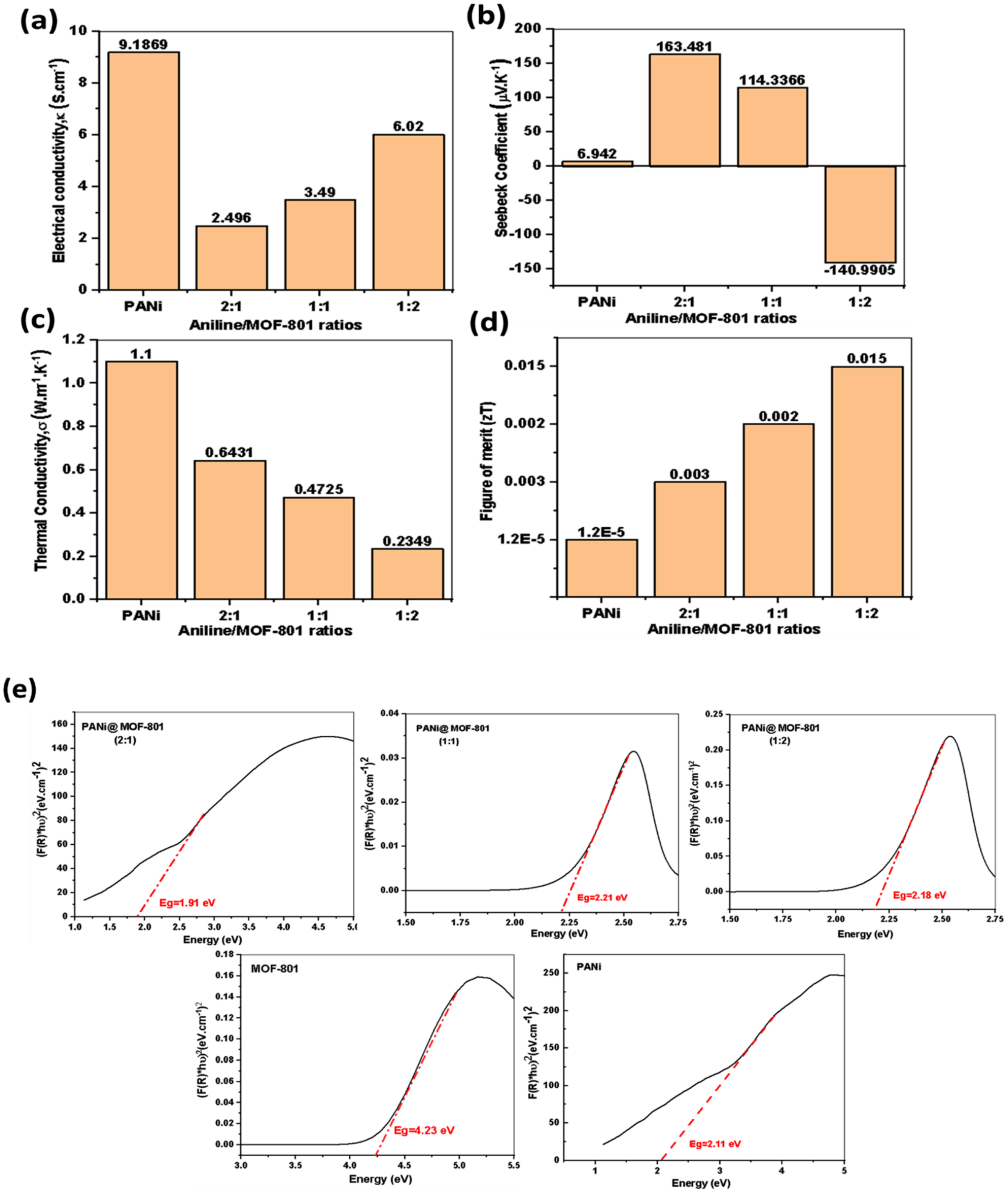
Composition dependent on (**a**) electrical conductivity, (**b**) Seebeck coefficient, (**c**) thermal conductivity, and (**d**) thermoelectric figure of merit of PANi and PANi@MOF-801 composites at room temperature, (**e**) Kubelka–Munk transformed reflectance spectra of MOF-801, PANi, and PANi@MOF-801 composites at room temperature.

According to the results of electrical transport measurements in Fig. 6a and Table 1, neat PANi approached electrical conductivity of 9.19 S cm^−1^ with carriers’ mobility and concentration of 1.02 cm^2^V^−1^ s^−1^ and 7.6 × 10^18^, respectively. Polyaniline transport characteristics are known to be disorder-dependent, resulting in various transport properties within the metal–insulator spectrum, as explained by numerous transport theories^33^. Emeraldine polyaniline is insulating, but its conductivity may be adjusted by doping from 10^–1^ to 10^2^ S cm^−1^ and more. When exposed to aqueous protonic or functionalized acids, the –N= sites become protonated while the number of electrons in the polymer chain remains constant, resulting in the conducting emeraldine salt form. The amorphous PANi structure was reported to impede efficient ion passage in the channels and rapid charge carrier transfer through hopping. As a result, limited carrier mobility manifests itself, resulting in low conductivity^33^.

**Table 1:** Mobility, carrier concentration, and power factor of PANi@MOF-801 composites.

Sample	Carrier mobility, cm^2^ V^−1^ s^−1^	Carrier concentration, cm^−3^	Eg, eV	PF, μWm^−1^ K^−2^	S, µV K^−1^	Type
PANi	1.2	7.6 × 10^18^	2.11	0.0438	6.942	p-Type
PANi@MOF-801 (2:1)	1.3	11.2 × 10^18^	1.91	6.6831	163.481	p-Type
PANi@MOF-801 (1:1)	2.01	24.9 × 10^18^	2.21	4.581	114.3366	p-Type
PANi@MOF-801 (1:2)	8.37	− 5.06 × 10^18^	2.18	12.325	− 140.9905	n-Type
MOF-801	–	–	4.23	–	–	

On the other hand, the electrical conductivity of MOF-801 due to its insulating nature is in the order of 10^–6^ S cm^−1^^11^. Generally, the connected rigid metal ions and redox-inactive organic ligands raise the energy barrier for electron transfer, making them electrical insulators. Upon incorporation of PANi as guest molecules, a remarkable increase in electrical conductivity and improving the concentration and the mobility of charge carriers are noticed, Fig. 6a and Table 1. The PANi@MOF-801 composites exhibited bulk electrical conductivity in the range of 2–7 S cm^−1^, which is 6 orders of magnitude higher than that of pristine MOF-801. Such an enhancement of the electrical transport properties could be mainly due to the synergetic interaction of conducting PANi guest chains with the host moieties of MOF, allowing good flow of electrons across the framework through the π–π interaction^12^. Our results seem to closely resemble the highly oriented growth of conducting PANi and its interfacial electronic interaction with aromatic ligands in MOF, developing percolating conducting paths and thereby bringing high electron mobility, Table 2. Meanwhile, it is also noted in Table 1 that the carrier concentration and mobility in the composites increased with MOF loading, matching the enhancement in the crystallinity of PANi by MOF loading.

**Table 2:** Summary of thermoelectric parameters for different reported MOFs.

MOF material	σ (Scm^−1^)	S (µVK^−1^)	κ (Wm^−1^K^−2^)	PF (µWm^−1^K^−2^)	zT	Refs.
PANi/Co-Ag-MOF	4.2*10^–2^	59.5	–	–	–	^12^
PANi/Co-MOF-Br	4.8*10^–2^	66.5	–	–	–	^12^
PANi/Co-MOF	5.9*10^–2^	46.3	–	–	–	^12^
UiO-66/Pan/PSS	2.1*10^–2^	− 17,780	–	664	–	^24^
Cu-BHT	2000	− 21	1.99	88.2	0.013	^41^
Cu_3_(HHTP)_2_	2.28*10^–3^	− 121.4	–	3.15*10^–3^	–	^42^
Ni_3_(HITP)_2_	58.8	− 11.9	0.21	0.833	1.19*10^–3^	^39^
Ni-PTC	9	47	0.2	1.988	0.003	^40^
Zn-HAB	0.86*10^–3^	200	–	3.44*10^–3^	–	^43^
TCNQ@Cu_3_(BTC)_2_	0.41*10^–2^	375	0.27	0.057	–	^13^
CNT @ZIF-67	825.7	55.6	4.1	255.6	0.02	^44^
PANi@MOF-801 (2:1)	2.49	163.481	0.6431	6.68306	3*10^–3^	This work
PANi@MOF-801 (1:1)	3.496	114.34	0.4725	4.58058	2*10^–3^	This work
PANi@MOF-801 (1:2)	6.2	− 140.99	0.2349	12.32447	15*10^–3^	This work

The UV–Vis diffuse reflectance spectra of the samples after the Kubelka–Munk treatment are shown in Fig. 6e. The intersection between the linear fit and the photon energy x-axis gives value to Eg. So, by this method the band gap of the prepared composites can be determined. From the data listed in Table 1, the band gap of MOF-801 is found to be 4.23 eV which indicates the insulating behavior of the MOF^34^. The band gap of the PANI is 2.11 eV^35^, electrical conductivity has been reported to rise in interactions between conjugated polymer and oxidants (p-type doping by an acceptor) which causes a reduction in the bandgap value (2.11 eV), by the addition of the MOF, the composites bandgaps slightly changed indicating the interaction between the guest polymer and the MOF.

The search for 3D MOFs to build Guest @MOF composites as potential thermoelectric materials is an overwhelming challenge, Table 2. The study of 3D MOFs as a thermoelectric material is still in its early stages. Xu et al. synthesized a 3D Co-based MOF using liquid 1-ethylpyridinium bromide (EtpyBr) and photosensitive AgNO_3_, for single crystal-to-single crystal transformation. They incorporated PANi with redox activity into MOF pores, resulting in three air-stable composites PANi/Co–MOF, PANi/Co–MOF–Br, and PANi/Co–Ag–MOF. These materials show moderate electronic conductivities and Seebeck coefficients of 5.9 × 10^−2^, 4.8 × 10^−2^, and 4.2 × 10^−2^ S cm^−1^ at 300 K, respectively. Their Seebeck coefficients were measured to be 46.3, 66.5, and 59.5 µV K^−1^ at 400 K^12^.

#### Seebeck coefficient

Achieving a high Seebeck coefficient, S, maybe the most intuitive method to increase ZT values as S influences ZT more significantly than other factors (ZT ∝ S^2^). The Seebeck coefficient values of all samples were measured at room temperature and the results are shown in Fig. 6b. As seen, PANi exhibited S value equals to + 6.9 µVK^−1^, with p-type transport behavior. The p-type behavior in PANI is thought to be caused by doping with low molecular weight anions such as chloride ions. When the Aniline/MOF ratios in the synthesis bath are 2:1 and 1:1, the S value increased to + 163.5 and + 114.3 µVK^−1^, respectively, and the composites maintained p-type transport behavior. With increasing the MOF ratio to double the aniline, that is 1:2, the composite exhibited S value of – 141 µVK^−1^ and hence, it turned to have n-Type transport behavior. In accordance with the presented results, it seems reasonable that MOF-801 shows a templating effect during the in-situ polymerization process, resulting in a more crystalline and extended polymer chain structure than the well-known amorphous and coil-like structure of neat PANi^24^. The elongated conformation increases the possibility of chloride ions passage across the transport channels and promotes carrier hopping through the framework, which explains the significant improvements in the S value of all synthesized nanocomposites, Table 1. This implies that the band gap is narrowing due to interactions between zirconium electron clouds (from the Zr-MOF) and nitrogen atoms (from PANi)^36^.

Furthermore, increasing the Zr-MOF component increases the carrier concentration and mobility of the composites, Table 1. When the aniline/MOF ratio is increased to 1:2, electron diffusion in the composite becomes simpler. Therefore, the PANi@MOF-801 composite has n-Type properties, resulting in negative Seebeck coefficients, as seen in Fig. 6b. It is believed that the observed improvement in Seebeck values accords with the Mott equation prediction, which states that better values are predicted with a decrease in carrier concentration (n) and an increase in carrier mobility (µ).

A significant π-π interaction between the MOF and the aromatic PANi structure has been documented in several research^37^. The Zr-MOFs transfer electrons to PANi through thermal excitation as a temperature gradient develops^38^. The Zr-MOF accumulates a high positive charge at the hot side after becoming immobile in its role as an electron donor to PANi. Due to PANi's role as an electron storage region at the cold end, a high concentration gradient of negative carriers’ forms, which ultimately produces large, negative Seebeck coefficients^24^. Interestingly, increasing MOF loading was found not only to increase the electrical transport properties, i.e., carrier mobility but also the porosity degree of the composites, inducing a higher Seebeck coefficient value. This enhancement in Seebeck value is attributed to the energy filtering effect that arises from the potential barriers that scattered cold carries with small path lengths such as phonons and electrons, allowing high energy electrons to pass through which increase the mobility, the thermopower, and reduce the thermal conductivity of the produced composites^39^. This trend proves that Seebeck coefficient and electrical conductivity are well-correlated despite the coupling effect of mobility and carrier concentration.

#### Thermal conductivity

To further illustrate the efficiency of the synthesized PANi@MOF-801 composites for use as thermoelectric materials, we investigated its thermal conductivity but only in perpendicular orientation (because of technical limitations associated with the measurements), Fig. 6c. The most notable property of MOFs is their extremely low thermal conductivity, which is among the lowest of any crystalline solid-state material. Thermal conductivity in solids around room temperature can be separated into electronic (κe) and lattice (κL) components. The former is commonly related to electrical conductivity by the Wiedemann–Franz law, κe = LσT, where the Lorenz number L is typically assumed to be 2.44*10^–8^ WK^−2^. MOF-801 is considered an insulator at room temperature, with a bandgap measured by UV-reflectance of 4.23 eV and very low electrical conductivity in the order of 10^–6^ S cm^−1^^4^. The experimentally reported bulk thermal conductivity in MOF-801, κL, is 0.1 W m^−1 ^K^−1^, while κe has a very low value, indicating that κL dominates the overall thermal transport in MOF-801^39^. Several causes may cause the low lattice thermal conductivity of MOFs. First, “phonon” refers to lattice vibrations carrying thermal energy that cannot bridge inherent empty pores. Second, phonon scattering is brought on by the heterogeneity of atomic masses and stiffness of bonds in MOFs.

Figure 6c shows the measured samples’ room temperature bulk thermal conductivity (κ). As can be seen, the recorded κ for the pristine PANi is 0.9 W m^−1^ K^−1^, which is considered higher than the reported values in the literature (0.2–0.3 W m^−1^ K^−1^)^4,39^. This large κ value may be attributed to the measurement technique or sample pressing. Upon the addition of different amounts from MOF-801, the κ is decreased to 0.64, 0.47, and 0.23 W m^−1^ K^−1^ for PANi@MOF-801 with Aniline /MOF ratios 2:1, 1:1, and 1:2, respectively. This can be explained by the fact that the total reduction in thermal conductivities is caused by the combined effect of low values for the framework and polymer chains, as well as the porous structure of the nanocomposite. Thus, the Zr-MOF-based in-situ polymerization approach presented here presents a unique chance to significantly enhance the amount of interfacial and phonon scattering at the interface, which would subsequently slow the rate of increase in thermal conductivity.

#### Power factor and figure of merit (zT)

The calculated PF of the PANi and its nanocomposites are listed in Table 1. The PF for the pristine polymer was the lowest at 0.044 μW m^−1^ K^−2^, and MOF-801 is an insulator. The PF for the PANi @ MOF 2:1, 1:1, and 1:2 is 6.7, 4.6, and 12.3 μW m^−1^ K^−2^, respectively. It is important to mention that the obtained PF values are among the highest recorded values for MOFs composites for thermoelectric applications^13,39,40^. This contributes favorably to the room temperature figure of merit (zT) calculated for the samples, as shown in Fig. 6d. All the PANi@MOF-801 nanocomposites showed an improved zT factor compared to pristine PANi and the MOF-801 itself. The best value was estimated to be 1.6 × 10^−2^ for the n-Type 1:2 Aniline/MOF ratio, which is five orders of magnitude higher than the 1.2 × 10^−6^ for pristine PANi. Several MOF materials studies reported enhanced zT values at room temperature in the range of 10^−3^–10^−4^^39,40^. Accordingly, our strategy that involves integrating PANi with MOF-801 succeeded in forming new types of composite thermoelectric materials with exceptionally high Seebeck coefficients and enhanced electrical conductivities and power factors. In addition, this design also suppresses the rate of increase in thermal conductivity, which in turn enhances the zT values of the composites. More importantly, the composite pellets' low-cost, lightweight, easily synthesized, better thermoelectric properties, and free-standing substrate composition. These structures are a perfect fit for using different MOF/polymer composites in thermoelectric applications.

Table 2 demonstrates that our results are comparable to most of the prepared composites of MOFs for thermoelectric applications. PANi@ MOF-801 recorded a higher electrical conductivity than PANi /Co-MOF with a higher Seebeck coefficient^12^. The power factor and zT-values of PANi@ MOF-801 composites in our work show a promising performance as room-temperature thermoelectric materials.

## Conclusion

In conclusion, MOF-801 and PANi nanocomposites for thermoelectric applications were successfully created with various ratios and p- and n-type conductivity properties. By using a simple hydrothermal method with Fumeric acid as a linker, the MOF-801 was initially created. The PANi@MOF-801 nanocomposites, however, were created by in-situ chemical oxidative polymerization of aniline in the presence of various Zr-MOF concentrations. It was confirmed through structural analysis using FTIR, XRD, TEM, and BET that PANi had successfully polymerized both on the surface and within the pores of the MOF.

On adding the PANi, it was found that both the Zr-MOF's electrical conductivity and Seebeck coefficient simultaneously improved. For the nanocomposites with an aniline/MOF ratio of 1:2, a Seebeck coefficient of − 141 µV K^−1^, a decent electrical conductivity of 6.2 S cm^−1^, and a negligibly low thermal conductivity of 0.24 W m^−1^ K^−1^were reported. This resulted in a room temperature figure of merit of 0.016 and an optimized power factor of 12.3 μW m^−1^ K^−2^. With the properties of highly crystalline porous structures, particularly metal–organic frameworks and conductive polymers, this work presents new potential for creating innovative thermoelectric materials.

## Data Availability

The data that support the findings of this study are available on request from the corresponding author, Asmaa Ebrahim [Asmaa.ebrahim@ejust.edu.eg].
